# PATHOLOGIC COMPLETE RESPONSE (YPT0 YPN0) AFTER CHEMOTHERAPY AND RADIOTHERAPY NEOADJUVANT FOLLOWED BY ESOPHAGECTOMY IN THE SQUAMOUS CELL CARCINOMA OF THE ESOPHAGUS

**DOI:** 10.1590/0102-672020180001e1405

**Published:** 2018-12-06

**Authors:** Nelson Adami ANDREOLLO, Giovanni de Carvalho BERALDO, Iuri Pedreira Filardi ALVES, Valdir TERCIOTI-JUNIOR, José Antonio Possato FERRER, João de Souza COELHO-NETO, Luiz Roberto LOPES

**Affiliations:** 1 Digestive Diseases Surgical Unit and Gastrocenter, Department of Surgery, School of Medical Sciences, State University of Campinas - UNICAMP , Campinas, SP, Brazil

**Keywords:** Esophagectomy, Esophageal carcinoma, Radiotherapy, Chemotherapy, Neoadjuvant therapy, Esofagectomia, Carcinoma do esôfago, Radioterapia, Quimioterapia, Terapêutica neoadjuvante

## Abstract

**Background:**

Esophageal squamous cell carcinoma is an aggressive neoplasia that requires a multidisciplinary treatment in which survival and prognosis are still not satisfactory. The complete pathologic response to neoadjuvant chemotherapy and radiotherapy is considered a good prognosis factor, and esophagectomy is indicated.

**Aim:**

Survival analysis of cases with pathologic complete response (ypT0 ypN0) to neoadjuvant chemotherapy and/or radiotherapy, submmitted to esophagectomy.

**Methods:**

Between 1983-2014, 222 esophagectomies were performed, and 177 were conducted to neoadjuvant treatment. In 34 patients the pathologic response was considered complete. Medical records of the patients were retrospectively reviewed regarding type of chemotherapy applied, amount of radiotherapy, interval between the neoadjuvant therapy and the surgery, body mass index; postoperative complications; hospital admission time and survival.

**Results:**

The average age was 55.8 years. Twenty-five patients were subjected to chemotherapy and radiotherapy, and nine to neoadjuvant radiotherapy. The total radiation dose ranged from 4400 until 5400 cGy. The chemotherapy was performed with 5FU, cisplatin, and carbotaxol, concomitantly with the radiotherapy. The esophagectomy was transmediastinal, followed by the cervical esophagogastroplasty performed on a average of 49.4 days after the neoadjuvant therapy. The hospital admission time was an average of 14.8 days. During the follow-up period, 52% of the patients submitted to radiotherapy and chemotherapy were disease-free, with 23.6% of them presenting more than five years survival.

**Conclusions:**

The neoadjuvant treatment followed by esophagectomy in patients with pathologic complete response is beneficial for the survival of patients with esophageal squamous cell carcinoma.

## RESUMO

**Racional:**

O carcinoma epidermoide do esôfago é neoplasia de natureza agressiva, que requer tratamento multidisciplinar e tem taxas de sobrevida e prognóstico ainda não satisfatórios. A resposta patológica completa à neoadjuvância com quimioterapia e radioterapia é considerada fator de bom prognóstico e a esofagectomia está indicada.

**Objetivo:**

Análise de sobrevida dos casos com resposta patológica completa (ypT0 ypN0) à neoadjuvância com quimioterapia e/ou radioterapia, submetidos à esofagectomia.

**Métodos:**

Entre 1983-2014, 222 esofagectomias foram realizadas e 177 foram submetidas ao tratamento neoadjuvante. Em 34 pacientes, a resposta patológica foi considerada completa. Os prontuários dos pacientes foram revisados retrospectivamente quanto ao tipo de quimioterapia aplicada, quantidade de radioterapia, intervalo entre a terapia neoadjuvante e a operação, índice de massa corporal (IMC), complicações pós-operatórias, tempo de internação hospitalar e sobrevida.

**Resultados:**

A idade média foi de 55,8 anos. Vinte e cinco pacientes foram submetidos a quimioterapia e radioterapia e nove à radioterapia neoadjuvante. A dose total de radiação variou de 4400 até 5400 cGy. A quimioterapia foi realizada com 5FU, cisplatina e carbotaxol, concomitantemente à radioterapia. A esofagectomia foi transmediastinal, seguida da esofagogastroplastia cervical realizada em média 49,4 dias após a terapia neoadjuvante. O tempo de internação hospitalar foi em média de 14,8 dias. Durante o período de seguimento, 52% dos pacientes submetidos a radioterapia e quimioterapia estavam livres de doença, com 23,6% apresentando sobrevida maior que cinco anos.

**Conclusão:**

O tratamento neoadjuvante seguido de esofagectomia, nos pacientes com resposta patológica completa, oferece benefícios na sobrevida de portadores de carcinoma epidermoide do esôfago.

## INTRODUCTION

Esophageal carcinoma is an aggressive cancer with low survival rate, the sixth in mortality and the eighth in incidence around the world. The esophageal squamous cell carcinoma (SCC) is the most common histological type, with incidence increasing with age, and peak in the seventh decade, associated in the majority of patients with smoking and alcohol consumption^31^ ^,^ ^48^. 

The neoadjuvant therapy followed by esophagectomy has been presenting promising results, and some studies reported increase of both the survival rate and the disease-free time, and also the reduction of its recurrence. The response to the neoadjuvant chemotherapy and radiotherapy varies among the patients, and they were considered responders or nonresponders. The responders might show complete or incomplete response. The tumoral complete pathologic response to the neoadjuvant therapy is determined by the absence of tumoral cells in the post-esophagectomy specimen, which is a good prognostic factor in the treatment of esophageal cancer^2^ ^,^ ^8^ ^,^ ^38^ ^,^ ^44^.

The esophagectomy associated with lymphadenectomy has been indicated and defended as a good alternative by several authors for patients with tumoral complete pathologic response to neoadjuvant, respecting an interval of approximately eight weeks between the end of the radiotherapy and the surgery^21^ ^,^ ^46^. This is a procedure that has high morbimortality rate, high cost, and should be performed in health services with intensive care units, besides pre- and postoperative care, and trained medical nursing staff that are used to perform it regularly^16,25,40, 41^.

The aim of this research was to analyze the results and survival rate of esophagectomy in patients with advanced esophageal squamous cell carcinoma and who had complete pathologic response to neoadjuvant chemotherapy and radiotherapy.

## METHOD

From 1983 until 2014, 222 esophagectomies were performed in the University Hospital of the University of Campinas (Unicamp), Campinas, SP Brazil, in patients with esophageal squamous cell carcinoma. The neoadjuvant treatment with chemotherapy and radiotherapy was indicated for 177 patients, from which 34 cases (19.2%) presented pathologic complete response (ypT0ypN0). These cases had complete absence of tumor cells in postoperative histopathological analysis.

The medical records of the patients were retrospectively reviewed regarding the type of chemotherapy, amount of radiotherapy, interval between the neoadjuvant therapy and the body mass index, postoperative complications, hospital admission time and survival rate. 

### Statistical analysis

The survival analysis was performed through the ANOVA (one-way) followed by the multiple comparison test of Dunnett, employing GraphPad Prism Version 6.00 for Windows, (GraphPad Software, La Jolla Califórnia, USA, www.graphpad.com), with signficance level of 5% (p< 0,05).

## RESULTS

The predominant location of the tumor was in the middle third of the esophagus in 24 cases out of 34 (70.6%); 28 of them were men with ages from 39 to 68 years (average 55.8 y).

Table 1 shows the distribution of patients regarding neoadjuvant and tumor location.

**TABLE 1:** Distribution of patients for neoadjuvant regarding the tumor location in the esophagus

Tumor location	Neoadjuvant				Total	
	RTX		RTX + QTX			
	n	%	n	%	n	%
Middle	9	26.5	15	44.1	24	70.6
Inferior	0	0	10	29.4	10	29.4
Total	9	26.5	25	73.5	34	100

RTX=exclusive radiotherapy; RTX + QTX=radiotherapy combined with chemotherapy

Nine patients received exclusive neoadjuvant radiotherapy, while others received chemoradiotherapy. The total radiation dose was above 4000 cGy in 88% of the patients during therapy, ranging from 4400-5400 cGy, divided in 25 to 30 sessions of 180 cGy per day. Table 2 shows the recommended doses of radiotherapy according to the tumor location.

**TABLE 2:** Distribution of patients regarding radiation dose according to the tumor location in the esophagus

Tumor location	Radiotherapy (cGy)							
	<4000		4001 to 4500		>4500		Total	
	n	%	N	%	n	%	n	%
Middle	4	11.8	9	26.5	11	32.3	24	70.6
Inferior	0	0	5	14.7	5	14.7	10	29.4
Total	4	11.8	14	41.2	16	47.0	34	100

The chemotherapeutic agents were cisplatin and 5FU (scheme Al Sarraf - for 21 patients) and carbotaxol and cisplatin (n=13); this treatment was combined with radiotherapy^1^ ^,^ ^11^ ^,^ ^12^ ^,^ ^15^ ^,^ ^23^ ^,^ ^43^.

The esophagectomy was performed during 30-60 days after the completion of the neoadjuvant therapy in most cases, with an average time of 49.4 days. The transmediastinal esophagectomy was followed by the cervical esophagogastroplasty and jejunostomy for postoperative enteral nutrition in all cases.The average BMI at the time of surgery was 20.9, ranging from 15.8 to 33.3. The hospital admission time was an average of 14.8 days.

The main general and local complications were: bronchopneumonia (n=6, 17.6%); urinary tract infection (n=2, 5.9%); cervical fistula of the esophageal-gastric anastomosis (n=7, 20.7%); and wound infection (n=4, 11.7%). Cervical fistulas were treated conservatively and all cases showed good recovery without the need for intervention.

Table 3 shows the survival time and follow-up of the two groups. Until the current date, all patients subjected to radiotherapy have died. However, in the group submitted to neadjuvant chemoradiotherapy, 11 patients have died up to 36 months after surgery (48%), and 13 (52%) keep being disease-free.

**TABLE 3:** Survival time and follow-up of patients with pathologic complete response to radiotherapy (RTX) and chemoradiotherapy (RTX + QTX)

Survival (months)	RTX	RTX + QTX	Total (%)
Up to 36	4	17	21 (61.7%)
36 to 60	3	2	5 (14.7%)
> 60	2	6	8 (23.6%)

**FIGURE 1:**
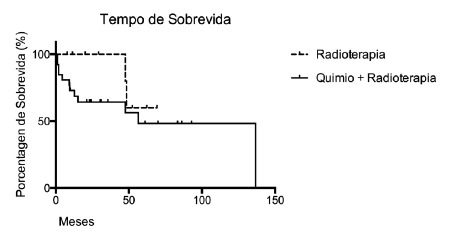
Survival curve of patients with pathologic complete response after radiotherapy and chemoradiotherapy

The Figure 1 shows the survival rate of patients with pathologic complete response to radiotherapy and chemoradiotherapy

Statistical analysis of the survival curves showed no statistical difference (p>0.05) between the treatments. However, the number of patients undergoing RTX + QTX with survival and follow-up of 60 months was higher compared to the ones submitted only to radiotherapy.

## DISCUSSION

The neoadjuvant therapy as a therapeutic strategy in esophageal squamous cell carcinoma has been used for many years^19^ ^,^ ^20^. This neoadjuvance may be only preoperative radiotherapy in the tumor area or, more recently, the use of associated radiotherapy and chemotherapy, thus affecting the tumor area and possible metastases^15^ ^,^ ^43^.

Several recent publications assess the role of neoadjuvant therapy in squamous cell carcinoma^2^ ^,^ ^17^ ^,^ ^20^ ^,^ ^36^ ^,^ ^39^ ^,^ ^44^ ^,^ ^47^, regarding the disease-free interval and survival. The PET-CT has been suggested as a method for this assessment, still with inconclusive results^2^ ^,^ ^17^ ^,^ ^20^ ^,^ ^36^ ^,^ ^39^ ^,^ ^44^ ^,^ ^47^. However, doubts on the benefits for global survival of patients still persist^14^ ^,^ ^35^ ^,^ ^45^. Furthermore, it is essential to separate patients with some response of tumor reduction to neoadjuvant therapy from those who do not exhibit any tumor reduction^4^ ^,^ ^9^.

The Response Evaluation Criteria in Solid Tumors scale (RCIST), commonly employed to evaluate tumor responses to neoadjuvant therapy, was not very feasible to be used due to the specificities of the esophagus and its injuries. Factors such as the esophageal tumor reduction being a late event, and the presence of fibrotic or necrotic tissue after the neoadjuvant made harder the use of computed tomography (CT) in order to medically estimate tumor reduction^3^.

In that scenario, the use of histopathology of post-surgery specimen in order to improve the accuracy of tumor reduction is suggested since it precisely identifies patients who actually had a tumor response to the neoadjuvant^6^ ^,^ ^30^. Patients with absence of tumor cells in the surgical specimen were defined as pathologic complete response holders, and presumably would have the greatest benefit in terms of overall survival^28^.

Some authors group the responders to neoadjuvant therapy in: a) patients who showed some reduction in tumor size in the surgical specimen as compared to the preoperative clinical period^18^; and b) patients who had complete absence of tumor cells in postoperative histopathological analysis^7^. Patients with pathologic complete response undoubtedly have the greatest benefit of neoadjuvant therapy due to the increase of survival rate and disease-free interval^10^ ^,^ ^13^ ^,^ ^32^ ^,^ ^34^ ^,^ ^42^. In addition, the complete pathological response is independent of the interval between esophagectomy and neoadjuvance^34^.

Stahl et al.^37^, in Germany in 2005, analysing the neoadjuvant therapy for esophageal cancer had already discovered that one third of patients have had pathologic complete response after preoperative chemoradiotherapy. They suggested that patients with locally advanced squamous cell carcinoma should be considered for neoadjuvant therapy and surgery.

Meredith et al.^22^, in USA in 2010, identified in a retrospective multicentric study, 262 patients who were subjected to neoadjuvant therapy and esophagectomy. From these ones, 106 patients (40.5%) had pathologic complete response, 95 (36,3%), pathologic partial response and 61 (23.3%), pathologic non-response to neoadjuvant therapy. The R0 resection rate was higher among patients with pathologic complete response (100%), compared to 94.7% in those with partial response (p=0.02), and 87.5% in those without response (p=0.0007). There were 15 (14.2%) recurrences in patients with pathologic complete response, 22 (23.7%) in those with partial response, and 17 (28.8%) in those without tumoral response (p=0.04). Patients with pathologic complete response had five years disease-free survival, and overall survival of 52% and 52%, respectively, compared with 36% and 38% in those with partial response and 22% and 19% in those without response (p<0.0001, p<0.0001). Therefore, the authors concluded that patients treated with neoadjuvant therapy with pathologic complete response have had higher rate of R0 resections, lower rate of recurrence, and increased survival.

Attempting to measure the benefit of the pathologic complete response on the survival rate, in 2012, Scheer et al.^32^ collected data from 22 previously published articles. According to their statistical analysis, the overall survival of patients with complete pathological response was 93.1%, 75.0%, and 50.0%, at 2, 3, and 5 years respectively. However, survival in the same periods was 36.8%, 29.0%, and 22.6% in patients with residual tumor (p<0.025). The average survival of patients with pathologic complete response was significantly higher than in those with residual tumor (p=0.011). In conclusion, the authors argue that the data suggest the likelihood of survival of patients with pathologic complete response is two or three times higher when compared with esophagectomy patients with residual tumor.

Orditura et al.^26^ in Italy in 2012, retrospectively reviewed 113 patients with esophageal cancer submitted to preoperative radiochemotherapy. According to them, the difference in survival between patients with pathologic complete response and patients with partial response or without response, after the neoadjuvant therapy, was statistically significant (p=0.0002, HR=0.21, 95% CI 0.18 to 0.60). They also state that, in the multivariate analysis, the pathologic complete response was one of the variables associated with the increase of overall survival. In conclusion, they said that patients with pathologic complete response obtained a significantly higher probability of survival after neoadjuvant therapy, compared to patients with partial response or without response.

Siddiqui et al.^33^ in USA in 2014, retrospectively analyzed a group of 106 patients who were submitted to neoadjuvant therapy and esophagectomy. They noticed the occurrence of pathologic complete response in 29% of cases (n=31) who obtained an overall survival of 52 months, which was much higher than the 31.2 months survival of the entire group. Furthermore, it was also higher than the 40 months survival of patients that had some tumor reduction, however without pathologic complete response.

An extensive review about neoadjuvant chemoradiotherapy performed by Smithers et al.^36^ in 2013, concluded about the role of the pathologic complete response: a) patients who have had a complete response after preoperative chemoradiotherapy had a better prognosis than those with partial pathological responses; b) patients with complete pathologic complete response after preoperative chemotherapy had improved survival rate, compared with patients without tumor response; c) pathologic complete response rates after preoperative chemoradiotherapy ranged from 13% to 49%, with no clear relation with histology (squamous and adenocarcinoma), radiation dose, chemotherapeutic agents (up to 2010) and interval between resection and end of neoadjuvant therapy; d) The pathologic complete response rates increased with the growth of the total radiation dose, the smaller treatment times and the younger patients with higher doses of chemotherapy; e) The pathologic complete response rates are higher after preoperative chemoradiotherapy, compared with preoperative chemotherapy.

The final case-by-case analysis of the results showed that 19.2% of the treated tumors had pathologic complete response. Comparing the survival rates of the two groups, no statistical significant difference was found. However, the survival curve of patients previously subjected to radiotherapy and chemotherapy indicated that more patients had more than five years survival^36^.

The literature review shows that the authors recommend esophagectomy in patients with pathologic complete response and those are the patients with better survival benefit with the neoadjuvant treatment. They emphasize that the esophagectomy should be indicated at health services with specialized medical teams and in hospitals with intensive care resources where this procedure is routinely done^9^ ^,^ ^10^ ^,^ ^11^ ^,^ ^14^ ^,^ ^18^ ^,^ ^22^ ^,^ ^27^ ^,^ ^28^ ^,^ ^32^ ^,^ ^34^.

O’Sullivan et al.^27^ reviewed the methods to classify tumor response to neoadjuvant treatments and concluded that the endoscopy, CT and PET-CT, are not sufficiently sensitive to confirm the complete response as reliable, and due to this it is essential to identify the biomarkers of the presence or absence of neoplastic disease in patients who can benefit from the esophagectomy. 

Bollschweiler et al.^5^ discuss the importance of developing predicting tumor response methods in order to identify responders before starting neoadjuvant treatments. Several retrospective studies employ molecular markers for response prediction, however, they are not clinically usable. Another issue is the assessment of tumor response after the neoadjuvant treatment protocol. A future prospect might be the combination of imaging techniques and special molecular markers for individualized therapy.

Considering the limited results of survival from surgical treatment in esophageal SCC, the neoadjuvant therapy consolidates itself as a good therapeutic strategy. Therefore, the group of patients who experienced pathologic complete response, as defined above, is the one with greater benefit from the neoadjuvant therapy.

## CONCLUSION

The neoadjuvant treatment followed by esophagectomy in patients with pathologic complete response is beneficial for the survival of patients with esophageal squamous cell carcinoma.
